# Construction of a Medical Micro-Object Cascade Network for Automated Segmentation of Cerebral Microbleeds in Susceptibility Weighted Imaging

**DOI:** 10.3389/fbioe.2022.937314

**Published:** 2022-07-20

**Authors:** Zeliang Wei, Xicheng Chen, Jialu Huang, Zhenyan Wang, Tianhua Yao, Chengcheng Gao, Haojia Wang, Pengpeng Li, Wei Ye, Yang Li, Ning Yao, Rui Zhang, Ning Tang, Fei Wang, Jun Hu, Dong Yi, Yazhou Wu

**Affiliations:** ^1^ Department of Health Statistics, College of Preventive Medicine, Army Medical University, Chongqing, China; ^2^ Department of Neurology, Southwest Hospital, Army Medical University, Chongqing, China; ^3^ Department of Medical Engineering, The 953 Hospital of the Chinese People’s Liberation Army, Shigatse, China; ^4^ Medical Big Data and Artificial Intelligence Center, Southwest Hospital, Army Medical University, Chongqing, China

**Keywords:** cerebral microbleed, medical micro-object, image segmentation, susceptibility weighted imaging, deep learning

## Abstract

**Aim:** The detection and segmentation of cerebral microbleeds (CMBs) images are the focus of clinical diagnosis and treatment. However, segmentation is difficult in clinical practice, and missed diagnosis may occur. Few related studies on the automated segmentation of CMB images have been performed, and we provide the most effective CMB segmentation to date using an automated segmentation system.

**Materials and Methods:** From a research perspective, we focused on the automated segmentation of CMB targets in susceptibility weighted imaging (SWI) for the first time and then constructed a deep learning network focused on the segmentation of micro-objects. We collected and marked clinical datasets and proposed a new medical micro-object cascade network (MMOC-Net). In the first stage, U-Net was utilized to select the region of interest (ROI). In the second stage, we utilized a full-resolution network (FRN) to complete fine segmentation. We also incorporated residual atrous spatial pyramid pooling (R-ASPP) and a new joint loss function.

**Results:** The most suitable segmentation result was achieved with a ROI size of 32 × 32. To verify the validity of each part of the method, ablation studies were performed, which showed that the best segmentation results were obtained when FRN, R-ASPP and the combined loss function were used simultaneously. Under these conditions, the obtained Dice similarity coefficient (DSC) value was 87.93% and the F2-score (F2) value was 90.69%. We also innovatively developed a visual clinical diagnosis system that can provide effective support for clinical diagnosis and treatment decisions.

**Conclusions:** We created the MMOC-Net method to perform the automated segmentation task of CMBs in an SWI and obtained better segmentation performance; hence, this pioneering method has research significance.

## 1 Introduction

Cerebral small vessel disease (CSVD) refers to a combination of clinical, imaging and pathological manifestations triggered by various types of small vessel and capillary lesions in the brain, with cerebral microbleeds (CMBs) being one of the main manifestations (Greenberg et al., 2009; Pantoni, 2010). CMBs have been proven to be a diagnostic indicator for a variety of cerebrovascular diseases, such as stroke, dysfunction, dementia and cognitive impairment. The number, distribution, and size of CMBs are important imaging-based indicators that are used for clinical diagnosis and treatment (Shuaib et al., 2019). For example, the lobar distribution of CMBs may be used to indicate the risk of cerebral amyloid angiopathy (Farid et al., 2017). For patients, improvements in CMB detection and segmentation accuracy are beneficial to reduce the psychological stress and economic burden caused by misdiagnosis. For physicians, the efficient detection and segmentation of CMBs can effectively improve efficiency, and it can help physicians fully grasp the optimal treatment time (Yakushiji, 2015). Automated segmentation of CMBs is beneficial to save the time of both physicians and patients, which in turn provides opportunities for monitoring and analysing numerous neurologic diseases.

CMBs are a type of brain parenchymal lesion that occur when small blood vessels and capillaries rupture. CMBs can appear as small ovoid (<10 mm in diameter) hypointense signals in susceptibility weighted imaging (SWI) sequences of magnetic resonance imaging (MRI) and are extremely representative clinical microlesions (Wardlaw et al., 2013; Smith et al., 2019). However, the automated segmentation of microlesions represented by CMBs is a more difficult and challenging clinical task because CMBs are widely distributed throughout the brain. They are not only extremely small but also share a high degree of visual similarity with CMB analogues (such as calcification, rust, and veins) (Myung et al., 2021). In addition, CMBs present a blooming effect on MRI images, meaning that the volume of CMBs increase with increasing echo time, and various acquisition settings may affect CMB sizes (Rashid et al., 2021). Therefore, there is a significant need to implement automated segmentation of CMBs in SWI sequences, which is difficult but possible. However, most of the previous studies are limited to CMB target detection (Dou et al., 2016; Liu et al., 2019; Al-masni et al., 2020; Li et al., 2021b; Myung et al., 2021; Rashid et al., 2021), and there is a lack of related research on the automatic segmentation of CMBs.

Aiming at the difficulty of automated segmentation of CMBs in SWI sequences, we proposed a new medical micro-object cascade network (MMOC-Net) to perform automated segmentation of CMBs. This network consists of two stages, which can consider global and local information, and can achieve better feature extraction accuracy (Hu et al., 2020). In the first stage, a modified U-Net module is used to segment the potential CMB regions. In the second stage, the final segmentation task is performed by using a full-resolution network (FRN) at each region of interest, and residual atrous spatial pyramid pooling (R-ASPP) is added to extract multiscale information.

The major contributions of our work are as follows:

1) Research perspective: Few related studies on the automated segmentation of CMBs have been performed thus far, and we provide a more reliable and effective automatic segmentation system for CMBs. Compared with previous studies, in this paper, millimetre performance enhancements are a point of focus. In addition, a new segmentation method for CMBs, which is of pioneering method with research significance, is proposed.
2) Research methods: We propose a new MMOC-Net method to perform segmentation. The FRN in the network can generate full-resolution features to boost the pixel-level segmentation performance. R-ASPP can extract multiscale image features based on the FRN to circumvent the risk of gradient explosion. The joint loss function can consider various evaluation metrics to ensure the best segmentation performance.
3) Evaluation metrics: We propose two evaluation indices, the Dice similarity coefficient (DSC) and the F2-score (F2), which are suitable for medical micro-objective segmentation. The DSC is the primary metric that is used to evaluate medical segmentation and can reflect the proportion of pixels correctly identified by the model. F2 can fully emphasize the importance of the sensitivity (SEN) on the premise of considering the precision (PRE). The research concept in this paper is in accordance with practical clinical needs and can be implemented to effectively reduce the rate of missed clinical diagnosis. The results of this study show that the method can perform well in terms of some important indices, such as DSC and F2.
4) Application value: Considering the lack of sufficient and available public datasets for CMB segmentation, we collect all available SWI images from 316 patients (625 pieces) with CMBs and use manual markers to obtain labels. To the best of our knowledge, this is currently the largest datasets used in automated CMB segmentation. It can also be applied to more segmentation tasks due to its good generalizability.

## 2 Related Works

The detection and segmentation of CMBs have always been the focus of clinical applications. The traditional CMB detection task needs to be visually performed with the aid of valid visual scoring scales, such as the microbleed anatomical rating scale (MARS) (Gregoire et al., 2009) or the brain observer microbleed scale (BOMBS) (Cordonnier et al., 2009). Traditional detection methods include technologies based on unified segmentation (Seghier et al., 2011), support vector machines (SVMs) (Barnes et al., 2011) or radial symmetry transformations (RSTs) (Bian et al., 2013; Kuijf et al., 2013). However, traditional methods suffer from low detection efficiency, poor accuracy, and high missed diagnosis rates and have been gradually replaced by deep learning methods.

In recent years, image processing methods based on deep learning techniques have been gradually proposed and promoted. Some studies on fully automated medical image detection and segmentation have been performed. Currently, the most traditional deep learning method is the convolutional neural network (CNN) (Havaei et al., 2017). CNN architectures use two paths to extract image features at various scales. The fully convolutional network (FCN), which is a tree-structured multitask network, was constructed with high efficiency for end-to-end network construction (Shelhamer et al., 2017). However, FCNs still suffer from difficulties in fine segmentation tasks. Ronneberger proposed a U-shaped convolutional network called U-Net, which performs well on various medical image segmentation tasks (Ronneberger et al., 2015). However, U-Net does not work well on medical micro target fine segmentation tasks.

There is a significant need to implement automated segmentation of CMBs in SWI sequences, which is difficult but possible. Myung et al. (2021) proposed a two-stage approach to conduct CMB detection based on the you only look once (YOLO) model, which achieved an SEN of 80.96%. Rashid et al. (2021) proposed DEEPMIR to detect CMBs and iron deposits, and an average SEN of between 84%–88% was achieved. Li et al. (2021b) used feature enhancement in CMB detection, and an SEN of 90.00% was achieved, suggesting that feature enhancement can be a helpful algorithm to enhance the deep learning model. Dou et al. (2016) performed CMB detection *via* 3D convolutional neural networks, and a sensitivity of 92.31% was achieved. Liu et al. (2019) presented a two-stage CMB detection framework that achieved a sensitivity of 93.50%. These studies demonstrate the potential of applying deep learning techniques for improving efficiency and accuracy in the diagnosis of CMB. However, these studies are mostly limited to target detection, and few relevant studies have been found to perform automatic CMB segmentation on SWI sequences.

## 3 Methodology

**FIGURE 1:**
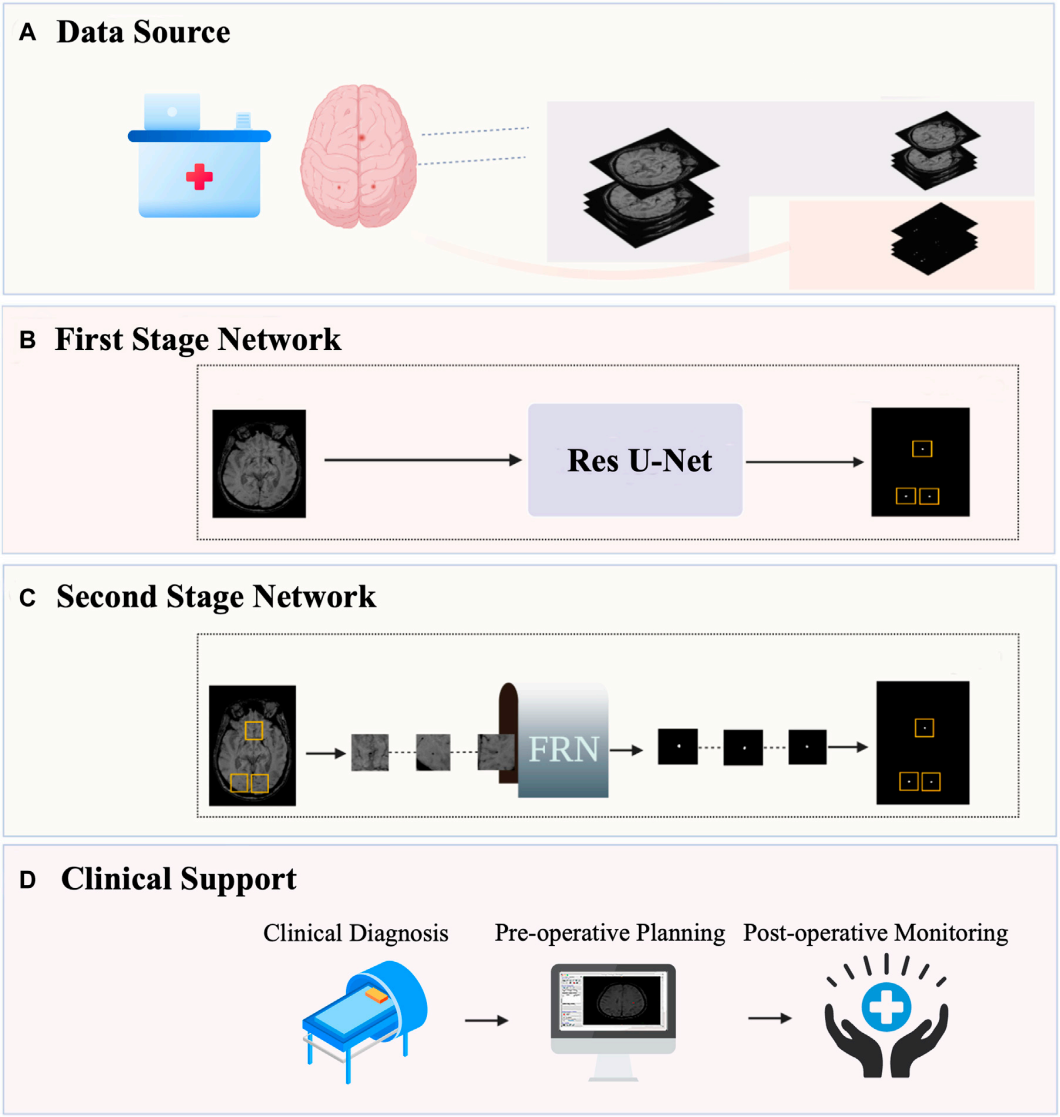
Overall framework of MMOC-Net. **(A)** Data collection and annotation process; **(B)** The first-stage coarse segmentation method uses Res U-Net to perform preliminary segmentation and identify the region of interest (ROI); **(C)** The second-stage fine segmentation method: FRN stands for full resolution network. The ROI regions are selected and input into the FRN to complete fine segmentation. **(D)** Automatic segmentation can obtain the distribution, quantity and size of lesions and support clinical decision-making.

Medical image segmentation is a challenging task in the field of computer vision. Because the segmentation target of CMB lesions is small and the effect of the single-step segmentation method is limited, we adopt the two-stage segmentation model MMOC-Net, which consists of coarse to fine segments to improve the detection and segmentation effect of lesions, and its overall framework is shown in Figure 1.

The first stage is coarse segmentation, and we utilize the improved U-Net to segment a whole original image. The focus of this stage is not on the PRE value of lesion contour segmentation but on the SEN value of lesion detection to ensure the full detection of potential lesions. The second stage is fine segmentation in which ROI regions of a certain size are selected, centred on the lesions detected in the first stage (including sizes of 16 × 16, 32 × 32, 64 × 64, and 128 × 128) and input into the FRN for local fine segmentation. This process ensures that segmentation contours are accurate, excludes false positives and increases the overall PRE in micro-object segmentation.

### 3.1 Data Source

Our data were obtained from cranial MR images of 316 patients with CMBs at the Department of Neurology, Southwest Hospital, Army Medical University, Chongqing, China. The images of each patient were composed of the original image and the corresponding manually segmented mask. The imaging data of each patient contained MRI scans of the brain with various sequences, including T1, T2 and SWI, of which the SWI sequence was used. Segmentation masks for all patients were jointly annotated by two experienced clinicians, providing a binary image containing manual segmentation for each subject to ensure the accuracy of the training data.

Considering pertinence and accuracy and ensuring a sufficient sample size and diversity of the sample distribution, we selected 1–4 scans from the complete images of each patient, for a total of 625 scans. These data were divided into two subsets with a ratio of 4:1. In other words, there were 500 pieces of training data and 125 pieces of validation data. This study was reviewed and approved by the hospital ethics committee under ethics number (B) KY2021173.

The intensities of MR images are unnormalized data, so we adopted the min-max normalization method to process the images for intensity values. In addition, to improve data utilization and model generalization efficacy, several data augmentation strategies were used: 1) A random intensity translation and scaling were applied across each channel with standard deviation (−0.2∼0.2). 2) A random rotation with a 30% probability of rotation and a rotation degree limited to 180° were used. 3) Random horizontal and vertical flips were performed with a 30% probability. 4) Random noise was added.

### 3.2 Model Construction

#### 3.2.1 First Stage Network

**FIGURE 2:**
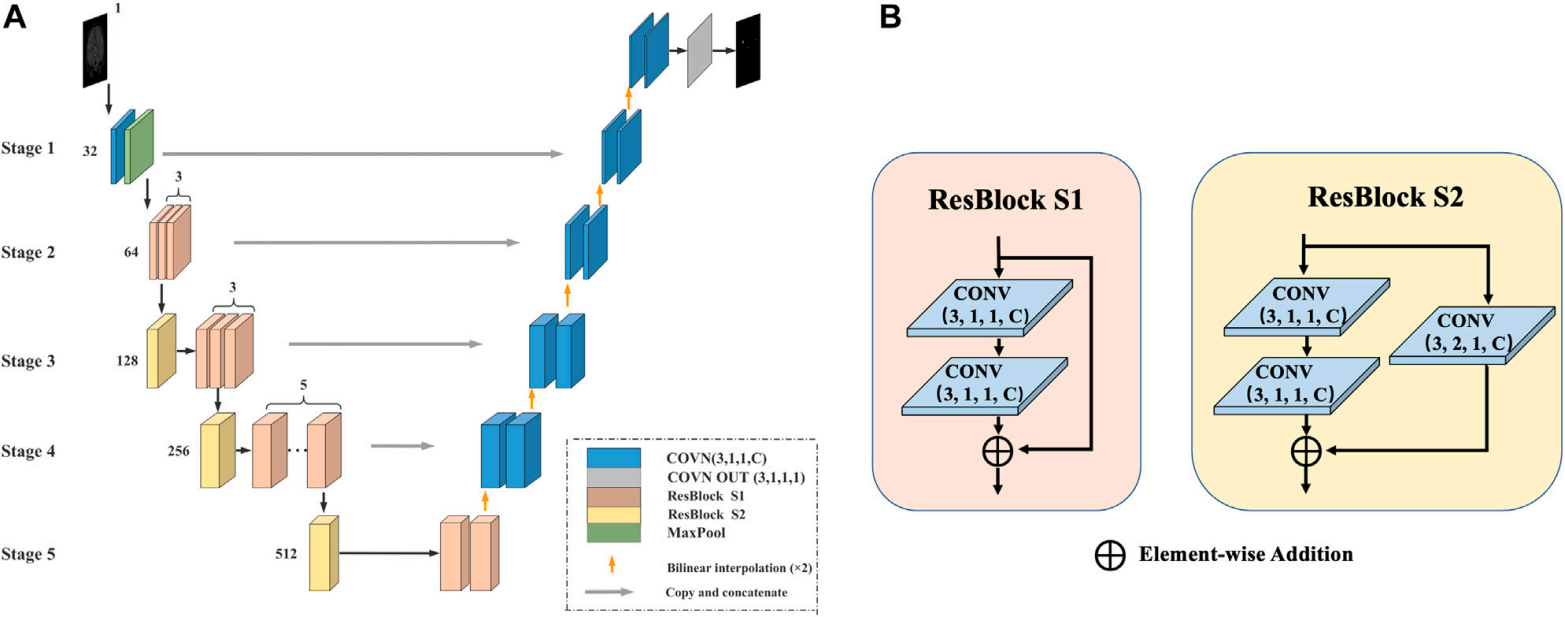
The first stage of the method. **(A)** U-Net model for coarse segmentation: The number represents the number of channels, the encoder is replaced with Res-Block during the encoding process, and the deconvolution is replaced with bilinear interpolation during the decoding process. **(B)** Res-Block S1/S2: The Res-Block S1 module contains two convolutional layers and uses shortcut connections to ensure the consistency of the gradient. The Res-Block S2 module adds a convolutional layer to the base of S1.

In the first stage, we use the improved U-Net model after pretraining to coarsely segment the CMB, as shown in Figure 2.

The U-Net model consists of an encoder (used for downsampling), a decoder (used for upsampling), and a skip connection and is divided into five stages. 1) Encoding process: The original encoder is replaced with a residual module (Res-Block), which can prevent the problem of vanishing/exploding gradients to provide a faster convergence rate and reduce the risk of overfitting. The output channel number of Stage 1 is 32. Stage 2 contains 3 Res-Block S1 modules, and the number of output channels is 64. Stage 3 contains 1 Res-Block S2 module and 3 Res-Block S1 modules, and the number of output channels is 128. Stage 5 contains 2 Res-Block S1 modules and 1 Res-Block S2 module, and the number of output channels is 512. 2) Decoding process: All deconvolutions are replaced with bilinear interpolation. The advantage is that no additional training parameters are needed, which can effectively reduce the model size and increase the running speed. 3) Skip connection: In each stage, a skip connection is used to fuse information in the encoding and decoding processes.

Our Res-Block is defined as:

$$y_{l}=h\left(x_{l}\right)+F\left(x_{l},W_{l}\right)$$ (1)

$${\text{x}}_{l+1}=f\left(y_{l}\right)$$ (2)

where $x_{l}$ and $x_{l+1}$ are the input and output of the *l* residual unit, *F* is the residual function, $W_{l}$ is the weight coefficient of the *l*th layer, $h\left(x_{l}\right)=x_{l}$ is the constant mapping, and f () represents the rectified linear unit (ReLU) activation function.

Based on the above information, the learning characteristics from *l* to *L* can be obtained as:

$$x_{L}=x_{l}+\sum_{l=1}^{L-1}F\left(x_{i},W_{i}\right)$$ (3)

Using the chain rule, the gradient of the reverse process can be found as:

$$\frac{\partial loss}{\partial x_{l}}=\frac{\partial loss}{\partial x_{L}}\times \frac{\partial x_{L}}{\partial x_{l}}=\frac{\partial loss}{\partial x_{L}}+\left(1+\frac{\partial }{\partial x_{L}}\sum_{l=1}^{L-1}F\left(x_{i},W_{i}\right)\right)$$ (4)

Here, *loss* is the loss of the learning process, and $\frac{\partial loss}{\partial x_{l}}$ is the gradient of the loss arriving at *L*. The number one represents the fact that a shortcut can propagate the gradient without decay, while the residual gradient on the other side needs to be passed through the weighted layer.

#### 3.2.2 Second Stage Network

**FIGURE 3:**
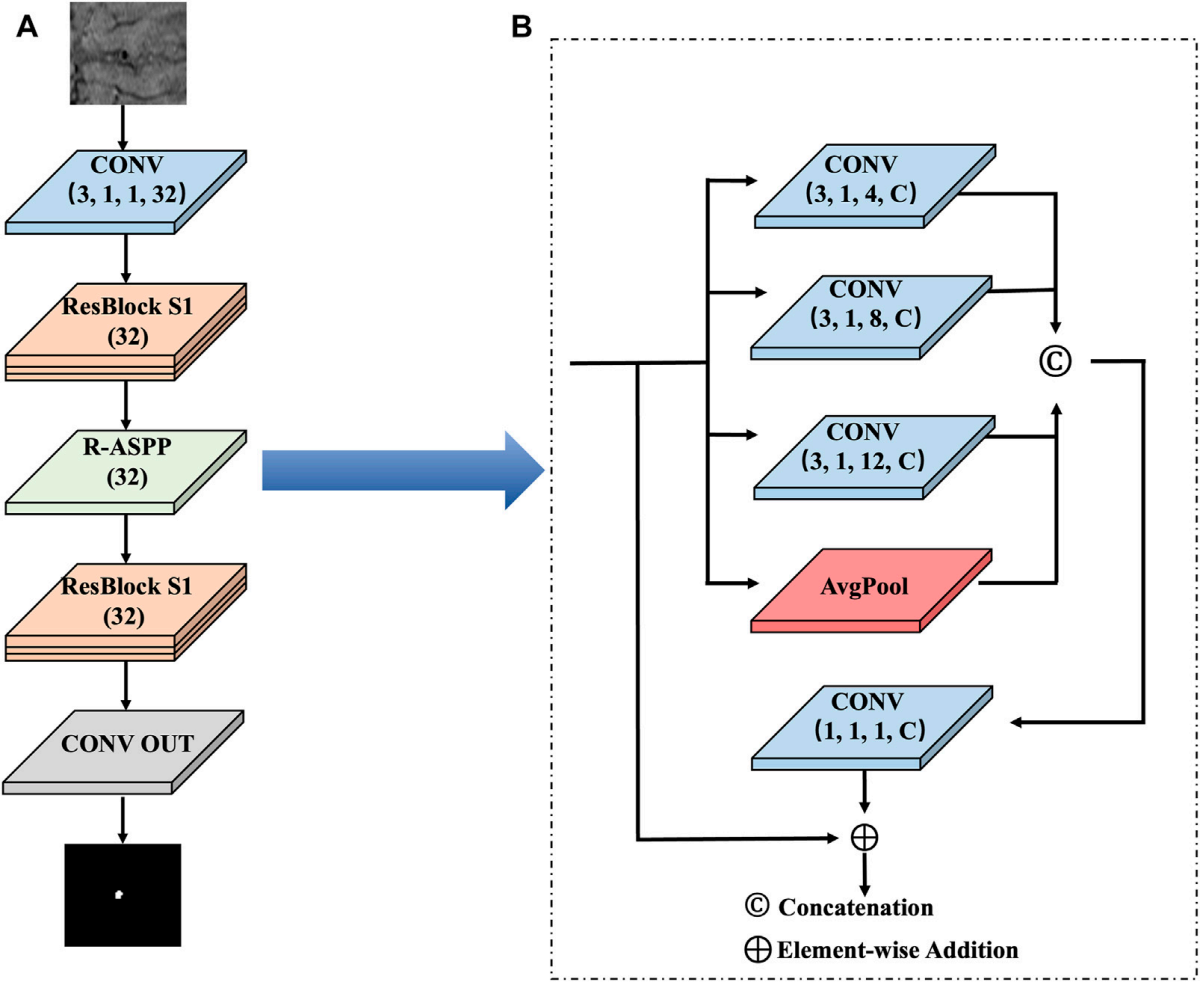
The second stage of the network architecture. **(A)** FRN framework: This framework removes the downsampling structure and always extracts image features at the original input resolution to effectively prevent lesion information loss. **(B)** R-ASPP module: In the middle of the FRN, we innovatively add the R-ASPP module.

In the second stage, we propose an FRN and added the R-ASPP module using a fine segmentation method to obtain the final segmentation result, as shown in Figure 3.

The FRN includes two convolutional layers, six residual modules and 1 R-ASPP module. We segment the target image patch centred on the region that is located by coarse segmentation. Then, we use the target image patch as the input of the FRN to focus on the precise segmentation of the target area without searching for the lesion on the entire image. After the target image patch is input to the first layer of convolution, a 32-channel feature map can be obtained, and then 3 Res-Block S1 modules are used to extract shallow features.

In the middle of the FRN, we innovatively add the R-ASPP module to extract multiscale features, the next 3 Res-Block S1 modules to extract deep features, and finally compress the features into one channel through CONV OUT to complete the result output. This module can perform parallel sampling of dilated convolutions with different sampling rates for a given input, and it can also prevent gradient disappearance or explosion.

In the R-ASPP module, feature maps are input into the dilated convolutions with dilation rates of 6, 8, 12, and 1 in the flat pooling layer. Their outputs are integrated and input into a convolution layer with a kernel size of 1 × 1 to obtain the number of channels consistent with that of the input feature map. The output of the previous convolutional layer and the input feature map are connected by shortcuts to obtain the result, and the output is $f_{\text{R}-ASPP}$ , which is defined as follows:

$$f_{ASPP}=K_{1}⊙\left(\sum_{i=1}^{3}K_{di}⊙f_{0}+AP\left(f_{0}\right)\right)$$ (5)

$$f_{R-APSS}=f_{ASPP}+h\left(f_{0}\right)$$ (6)

where ⊙ denotes the convolution operation, *f*_0_ is the input feature map, AP denotes the average pooling operation, *K*_1_ represents that the size of the convolution kernel is 1 × 1, *K*_*di*_ represents that the size of the convolution kernel is 3 × 3, the dilation rate is d_*i*_ = (4,8,12), and *h* represents the identity mapping of the shortcut connection.

### 3.3 Modified Loss Function

Due to the imbalance between the pixels of CMBs and the background, binary cross entropy (BCE) loss and dice loss (DL) are taken as the loss functions. In addition, we creatively add the SEN loss function. The functions and advantages of each loss function are discussed below.

BCE is derived from the maximum likelihood function under the condition of a Bernoulli distribution and has been widely used in object classification and image pixel-level segmentation. BCE is used to measure the difference between two probability distributions of a given random variable or an event set. The smaller the value is, the smaller the difference between the two probability distributions. BCE can be defined as follows:

$$L_{BCE}\left(\hat{y},y\right)=-\frac{1}{N}\sum_{i=1}^{N}[y_{i}\log\left({\hat{y}}_{i}\right)+\left(1-y_{i}\right)\log\left(1-{\hat{y}}_{i}\right)]$$ (7)

DL is more suitable for unbalanced tasks such as medical image segmentation. The DL loss value represents the predictive performance of the trained model. DL can be defined as:

$$L_{Dice}\left(\hat{y},y\right)=1-\frac{2\sum_{i=1}^{N}{\hat{y}}_{i}y_{i}}{\sum_{i=1}^{N}{\hat{y}}_{i}+\sum_{i=1}^{N}y_{i}}$$ (8)

In the CMB segmentation task, there is an imbalance between the foreground and the background. In terms of the clinical needs of the CMB segmentation task, an improvement in SEN is more important than an improvement in PRE. Therefore, we propose an SEN loss function whose value represents the missed diagnosis rate; it can be defined as:

$$L_{Sen}\left(\hat{y},y\right)=1-\frac{\sum_{i=1}^{N}{\hat{y}}_{i}y_{i}}{\sum_{i=1}^{N}y_{i}}$$ (9)

Here, $\hat{y}$ and y are the model prediction result and the real label image, N is the number of pixels in the input image, and ${\hat{y}}_{i}$ is the value of pixel i in $\hat{y}$ .

In conclusion, BCE can improve the stability of model training, DL can make the model prediction results more closely resemble the expected value, and the SEN loss function can improve the SEN of model training. Therefore, we combine three types of loss functions to construct the total loss function as follows:

$$L_{total}=L_{BCE}+L_{Dice}+\lambda L_{Sen}$$ (10)

Here, the value of *λ* can affect the degree of the influence of the SEN loss value, and the larger the value is, the higher the weight of the influence of SEN. We determine the optimal value of *λ* after many pre-experiments. The focus of the coarse segmentation stage is to detect as many potential lesions as possible, so we set *λ* = 10. The focus of the fine segmentation stage is to balance SEN and PRE, so we set *λ* = 1. In model training, the total loss function value gradually decreases until it becomes stable, and the training model with the best prediction performance can be obtained.

### 3.4 Training Details

The training process is accelerated using a GPU, and the details of the running environment are shown in Table 1.

**TABLE 1:** Summary of the experimental environment.

Software/Hardware	Model/Parameter
CPU	Intel Xeon (R) Gold 6246R CPU @ 3.30 GHz
GPU	NVIDIA Tesla V100
RAM	256 GB
Hard disk	1 TB
System	Cent Os 8
Framework	Python 3.7, Pytorch 1.3
Module	SimpleITK, Nibabel, TorchVison, Scipy, Numpy, etc.

In the coarse segmentation model training stage, all images are scaled to 352 × 448, and the input size of the ROIs in the fine segmentation model is 32 × 32 pixels. This design ensures that the lesion is of a detectable size while not exceeding the operating memory limit due to the image being too large. Both stages use the Adam optimizer to update the network weights, and the initial learning rate *η* is 0.0002.

During the training process, we also employ early stopping and cross-validation strategies, aiming to improve the persuasiveness of the experimental results and prevent the occurrence of overfitting during training. In each fold of the training set, we use early stopping and five-fold cross-validation strategies to select the number of epochs and the hyperparameters. We set the maximum number of data training iterations to 500 epochs and the patch size to 6. We also use the gradient checkpoint operation of PyTorch to reduce memory consumption.

### 3.5 Evaluation Metric

Confusion matrices are often used to evaluate the classification or segmentation tasks in deep learning, and $E_{TP}$ , $E_{TN}$ , $E_{FP}$ , and $E_{FN}$ are used to represent the number of pixel points in the true-positive, true-negative, false-positive and false-negative areas, respectively. The SEN, PRE, DSC, F2, Jaccard similarity coefficient (JSC) and Matthew correlation coefficient (MCC) are used to quantitatively evaluate the network segmentation results. The range of values for each index is (0–1), and a larger value represents a better segmentation effect.

SEN and PRE are important measures of missed diagnosis and misdiagnosis, respectively. Missed diagnosis and misdiagnosis are pairs of contradictions, and it is often impossible to completely prevent them. In actual clinical diagnosis and treatment, the incidence of a missed diagnosis of CMBs is significantly higher than that of a misdiagnosis, and the consequences of a missed diagnosis are far greater than those of a misdiagnosis. The occurrence of a missed diagnosis may incur extremely high treatment risks and treatment costs, which cause irreversible clinical consequences. Even if a misdiagnosis occurs, subsequent diagnosis and treatment can be implemented, and serious consequences rarely occur. Therefore, it is more important to reduce the risk of missed diagnosis in CMB diagnosis. SEN and PRE can be expressed as:

$$SEN=\frac{E_{TP}}{E_{TP}+E_{FN}}$$ (11)

$$PRE=\frac{E_{TP}}{E_{TP}+E_{FP}}$$ (12)

The DSC is a mask similarity metric that measures how similar a predicted image is to the pixel spots of the ground truth. DSC describes the degree of similarity by evaluating the ratio of the area of the overlapping part of the two ensembles to the total area and is calculated as:

$$DSC=\frac{2\left|{ℜ}_{a}⋂{ℜ}_{b}\right|}{\left|{ℜ}_{a}\right|+\left|{ℜ}_{b}\right|}$$ (13)

Here, ${ℜ}_{a}$ and ${ℜ}_{b}$ represent the image area set and ground truth value set segmented according to the DSC∈ (0,1) algorithm; the larger the value is, the better the fit between the model segmentation area and the real area.

The F-score comprehensively weighs the PRE and SEN indicators. F2 is a special case of the F-score, and it focuses more on the SEN indicator. Our optimization principle ensures a high SEN value under the premise of ensuring that the PRE value is acceptable. This design is intended to reduce the clinical missed diagnosis rate as much as possible under the premise of ensuring a certain diagnostic accuracy rate. Therefore, the definition of F2 is more in line with the needs of this research and can be defined as:

$$F_{2}=5\cdot \frac{PRE\cdot SEN}{\left(4\cdot PRE\right)+SEN}$$ (14)

We take the optimization of DSC and F2 as the primary goal and take DSC and F2 as the primary evaluation indices. To fully evaluate the effect of segmentation, we also introduce JSC and MCC as secondary evaluation indicators; they can be expressed as

$$JSC=\frac{E_{TP}}{E_{TP}+E_{FN}+E_{FP}}$$ (15)

$$MCC=\frac{E_{TP}\cdot E_{TN}-E_{FP}\cdot E_{FN}}{\sqrt{\left(E_{TP}+E_{FP}\right)\left(E_{TP}+E_{FN}\right)\left(E_{TN}+E_{FP}\right)\left(E_{TN}+E_{FN}\right)}}$$ (16)

The raincloud plot is a data visualization tool that provides more statistical information. It draws on the advantages of a variety of traditional statistical graphs and visualizes the original data, probability density and key statistical information (i.e., the median, average, and confidence interval) (Allen et al., 2019). From the term raincloud, “rain” represents the original data lattice, and “cloud” represents the data distribution. Box plots, central tendency information and error bar information are also added to the figure to further improve the statistical information. In a raincloud plot, redundant mirroring probability distributions are replaced with box plots and original data points and can provide important information such as data relationships and data distributions.

Medical datasets often have the problem of unbalanced categories, so we additionally choose the P-R curve to intuitively reflect the segmentation effect of the model. SEN and PRE can be used to draw precision-recall (P-R) curves, and the model performance can be better judged by the inclusion relationship of the P-R curve. The area under the P-R curve can be defined as the average PRE (AP) value. The higher this value is, the better the overall classification effect of the pixels.

## 4 Results

### 4.1 MMOC-Net Segmentation Results

In Table 2, the segmentation results are presented for the validation set using an ROI size of 32 × 32. An average DSC value of 87.93% for five-fold cross-validation and an average F2 value of 90.69% were achieved.

**TABLE 2:** Segmentation performance (%).

Metrics	Fold 1	Fold 2	Fold 3	Fold 4	Fold 5	Mean
DSC	84.42 ± 14.55	90.84 ± 7.38	87.76 ± 8.19	88.72 ± 10.18	87.85 ± 11.19	87.93 ± 10.30
F2	86.65 ± 14.95	92.88 ± 6.10	90.75 ± 7.67	91.38 ± 7.94	91.70 ± 6.21	90.69 ± 9.39
SEN	89.76 ± 17.66	95.34 ± 6.79	94.74 ± 8.46	95.03 ± 8.81	94.50 ± 11.02	93.87 ± 10.55
PRE	79.68 ± 21.08	86.74 ± 12.89	81.74 ± 15.67	83.20 ± 15.52	82.07 ± 16.65	82.69 ± 16.36
JSC	70.07 ± 21.43	82.78 ± 12.23	77.42 ± 14.36	78.96 ± 14.55	78.32 ± 15.45	78.11 ± 15.60
MCC	83.61 ± 16.01	90.92 ± 8.19	87.51 ± 7.38	89.16 ± 8.96	87.34 ± 20.63	87.72 ± 12.23

**FIGURE 4:**
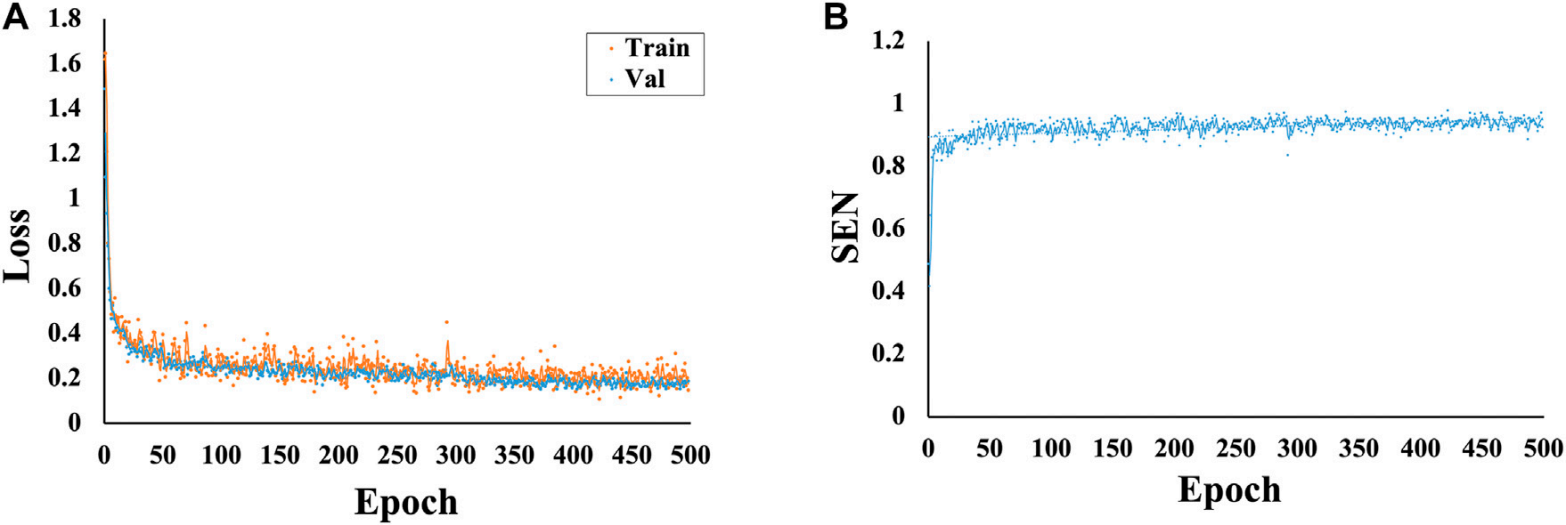
Variations in the loss and SEN values at different epochs. **(A)** Variations in the loss values at different epochs. **(B)** Variations in the SEN values at different epochs.

The total loss values and SEN changes at each epoch of the model are plotted and detailed in Figure 4. The results show that as the number of iterations increases, the total loss value decreases, the SEN value increases, and the convergence of our model is fast.

### 4.2 Effectiveness Comparison of Region of Interest Sizes

To investigate the effect of the ROI size on model performance, we compare the segmentation results of the four ROI sizes. The results show that the best segmentation results can be obtained when the size is 32 × 32. A DSC value of 87.93% and an F2 value of 90.69% were achieved under these conditions, as detailed in Table 3. Therefore, a size of 32 × 32 is adopted for each ablation combination in the later text.

**TABLE 3:** Comparison of the four ROI size segmentation performance results (%).

Metrics	16 × 16	32 × 32	64 × 64	128 × 128
DSC	86.09 ± 11.98^b^	**87.93 ± 10.30** ^a^	85.62 ± 11.92	85.23 ± 12.02
F2	89.13 ± 10.83	**90.69 ± 9.39** ^a^	90.24 ± 9.90^b^	89.83 ± 10.42
SEN	91.63 ± 12.47	**93.87 ± 10.55** ^b^	93.88 ± 11.19^a^	93.15 ± 13.24
PRE	83.82 ± 16.60^a^	**82.69 ± 16.36** ^b^	80.89 ± 17.06	80.43 ± 17.62
JSC	77.29 ± 16.09^b^	**78.11 ± 15.60** ^a^	76.49 ± 16.46	75.78 ± 17.34
MCC	86.93 ± 10.72^b^	**87.72 ± 12.23** ^a^	86.57 ± 10.54	86.20 ± 10.69

a ranked first.

b ranked second.

Note: Bold values represent the best results in the table.

**FIGURE 5:**
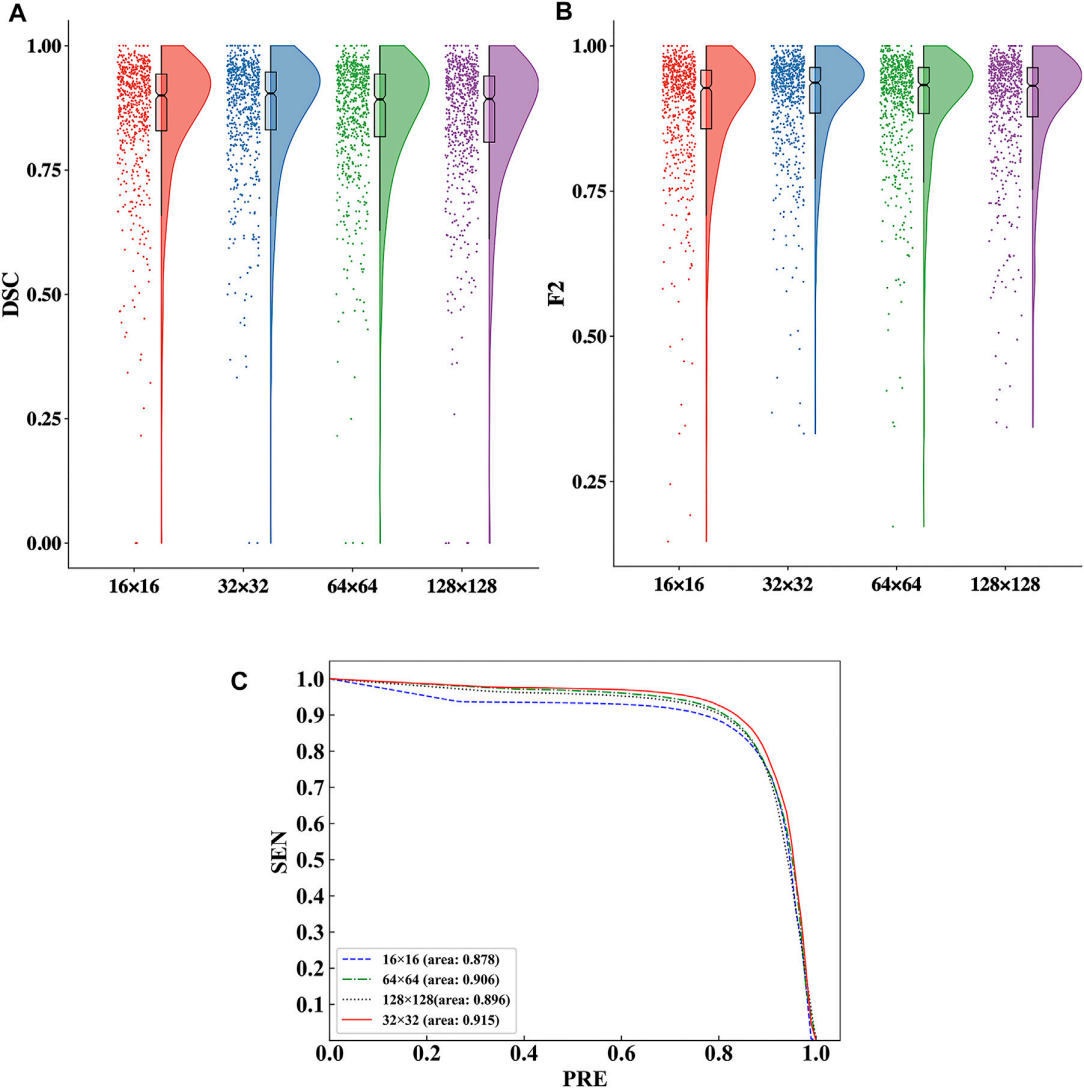
Comparison of the segmentation performance for the four ROI sizes. **(A)** Comparison of the DSC values in the raincloud plot for the four ROI sizes. **(B)** Comparison of the F2 values in the raincloud plot for the four ROI sizes. **(C)** Comparison of the P-R curves of the segmentation performance for the four ROI sizes.

The raincloud plots of DSC and F2 of the four ROI sizes are presented in Figures 5A,B; from these plots, we select the best ROI size. The results based on the raincloud plot for the 32 × 32 size have a better distribution of data, and the DSC and F2 values are closer to the high-score region. For the box plot, an overall segmentation accuracy improvement is evident with dimension a as well as the median and mean numbers of DSC and F2. Therefore, 32 × 32 is the optimal size for capturing image features with optimal segmentation and stability. The P-R curves of the four ROI sizes are presented in Figure 5C, and the results show that the inclusion relationship of the 32 × 32 size is significantly better than those of the other sizes, and the area under the curve is the largest (0.915).

**FIGURE 6:**
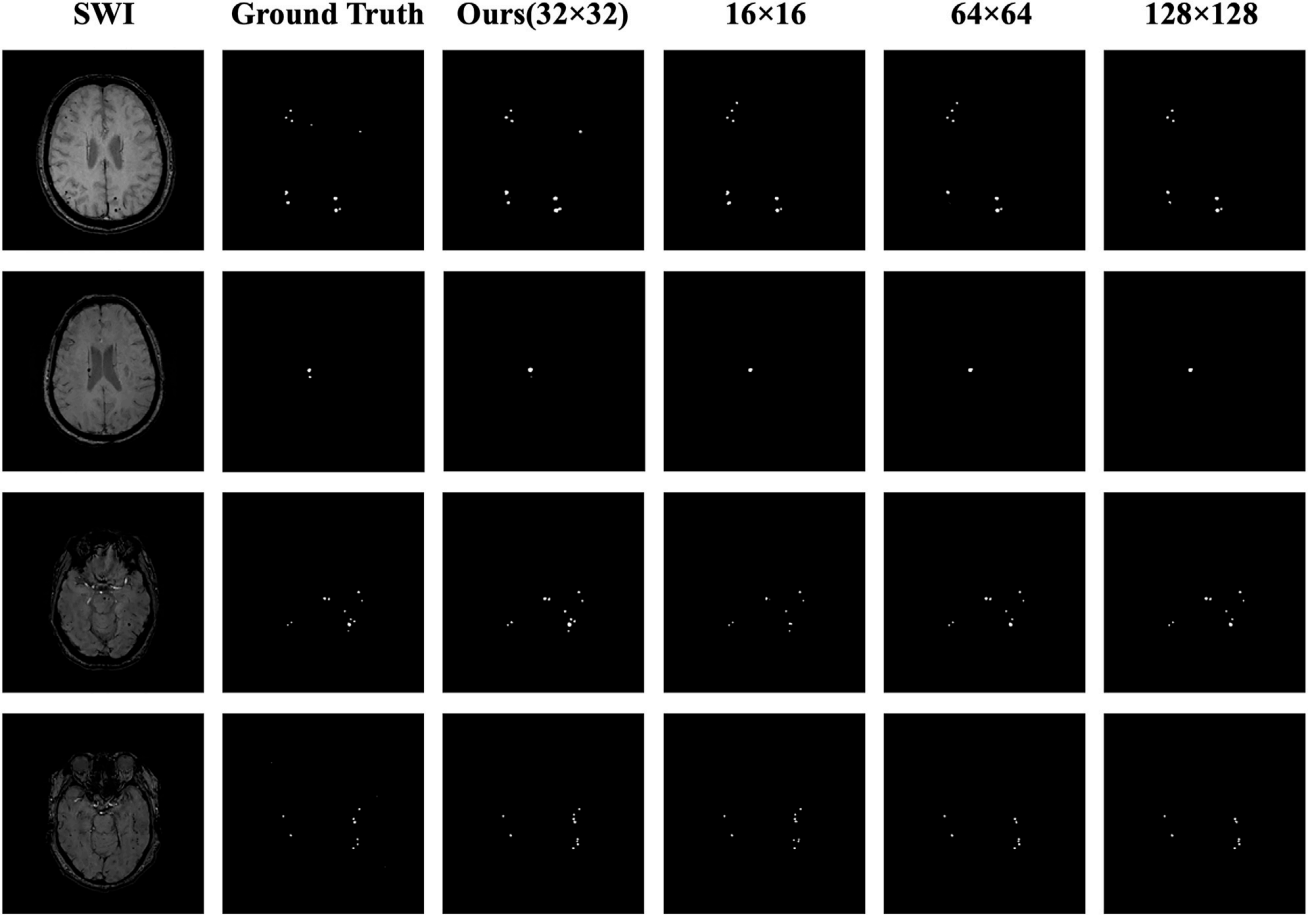
Comparison of the masks for the four ROI sizes. The columns from left to right represent the original image, the ground truth, the 32 × 32 size (ours), the 16 × 16 size, the 64 × 64 size, and the 128 × 128 size; the rows from top to bottom represent case Nos. 1–4, respectively. In case 1, the lesion in the upper right corner was correctly predicted by using the 32 × 32 size, while using the other three sizes, the diagnosis was missed or a misdiagnosis was made. In case 2, the small lesion in the lower right corner was correctly predicted by using the 32 × 32 size, whereas using the other three sizes, the diagnosis was missed. In case 3, the number and distribution of the lesions were correctly predicted using the 32 × 32 size, whereas using the other three sizes, the diagnosis was missed. In case 4, the result using the 32 × 32 size was closest to the number and distribution of the ground truth, while using the other three sizes resulted in missed diagnoses or made misdiagnoses.

We visually display the segmentation results of the four ROI sizes to contrast the segmentation effect of various ROI sizes, as shown in Figure 6. When the 32 × 32 size is utilized, the highest DSC value is achieved, and the segmentation details are closer to the ground truth than those of the other sizes. The apparent risks of missed diagnosis and misdiagnosis are lower and have the best segmentation performance.

### 4.3 Effectiveness Comparison of Ablation Combinations

To verify the validity of the components implemented in our method, we compare the segmentation performance of different fusion combinations. The results are compared separately by removing each segment, and the results show that MMOC-Net achieves the best segmentation results, with a DSC value of 87.93% and an F2 value of 90.69%. Table 4 for details.

**TABLE 4:** Comparison of the effectiveness of the four ablation combinations (%).

Metrics	W/o FRN	W/o R-ASPP	W/o L-SEN	MMOC-Net(Ours)
DSC	79.76 ± 11.32	85.84 ± 11.40	87.10 ± 10.91^b^	**87.93 ± 10.30** ^a^
F2	88.73 ± 8.50^b^	89.19 ± 9.93	88.19 ± 10.57	**90.69 ± 9.39** ^a^
SEN	96.12 ± 8.96^a^	92.00 ± 11.64	89.20 ± 12.77	**93.87 ± 10.55** ^b^
PRE	68.89 ± 15.27	82.91 ± 16.39^b^	87.52 ± 15.41^a^	**82.69 ± 16.36**
JSC	67.10 ± 14.86	76.71 ± 15.40	78.60 ± 15.01^a^	**78.11 ± 15.60** ^b^
MCC	81.41 ± 9.72	86.70 ± 10.24	87.77 ± 9.98^a^	**87.72 ± 12.23** ^b^

a ranked first.

b ranked second.

Bold values represent the best results in the table. W/o, without; FRN, full-resolution network; R-ASPP, atrous spatial pyramid pooling; L-SEN, Loss of sensitivity.

**FIGURE 7:**
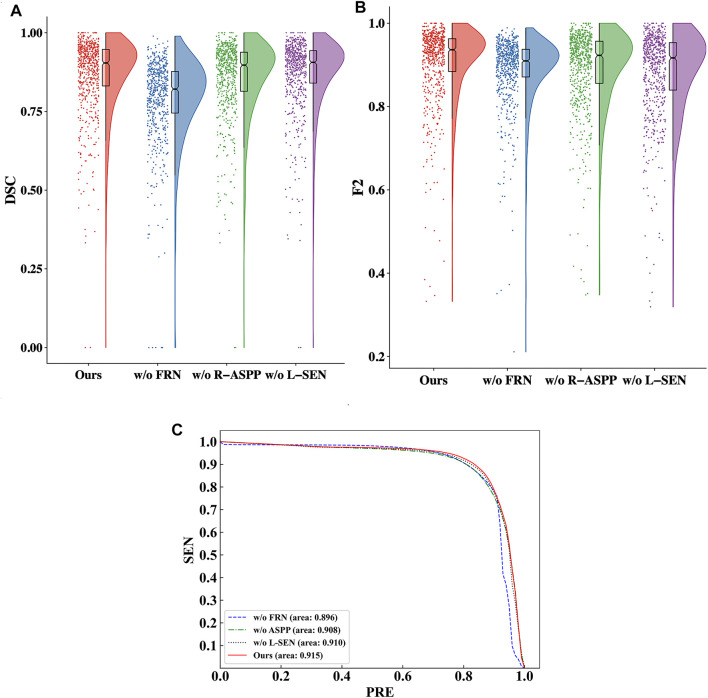
Comparison of the effectiveness of the four ablation combinations. **(A)** Comparison of the DSC values in the raincloud plot of the ablation combinations. **(B)** Comparison of the F2 values in the raincloud plot of the ablation combinations. **(C)** Comparison of the P-R curves of the segmentation performance results of the ablation combinations.

We utilize raincloud plots to compare the effectiveness when the four ablation combinations are chosen and aim to retrieve the best ablation combination, as shown in Figures 7A,B. In the raincloud plot, the data distribution of DSC and F2 in MMOC-Net is significantly better than those of the other combinations, and most of them are distributed in high-score regions. In the boxplot, the overall effect of the full MMOC-Net is increased, for both the medians and means of DSC and F2. Therefore, using MMOC-Net, including FRN, R-ASPP and the joint loss function, a better segmentation effect and stability can be obtained. The P-R curves of the four ablation combinations are shown in Figure 7C. The results show that the inclusion relationship of MMOC-Net is significantly better than those of the other combinations and has the largest area under the curve (0.915).

**FIGURE 8:**
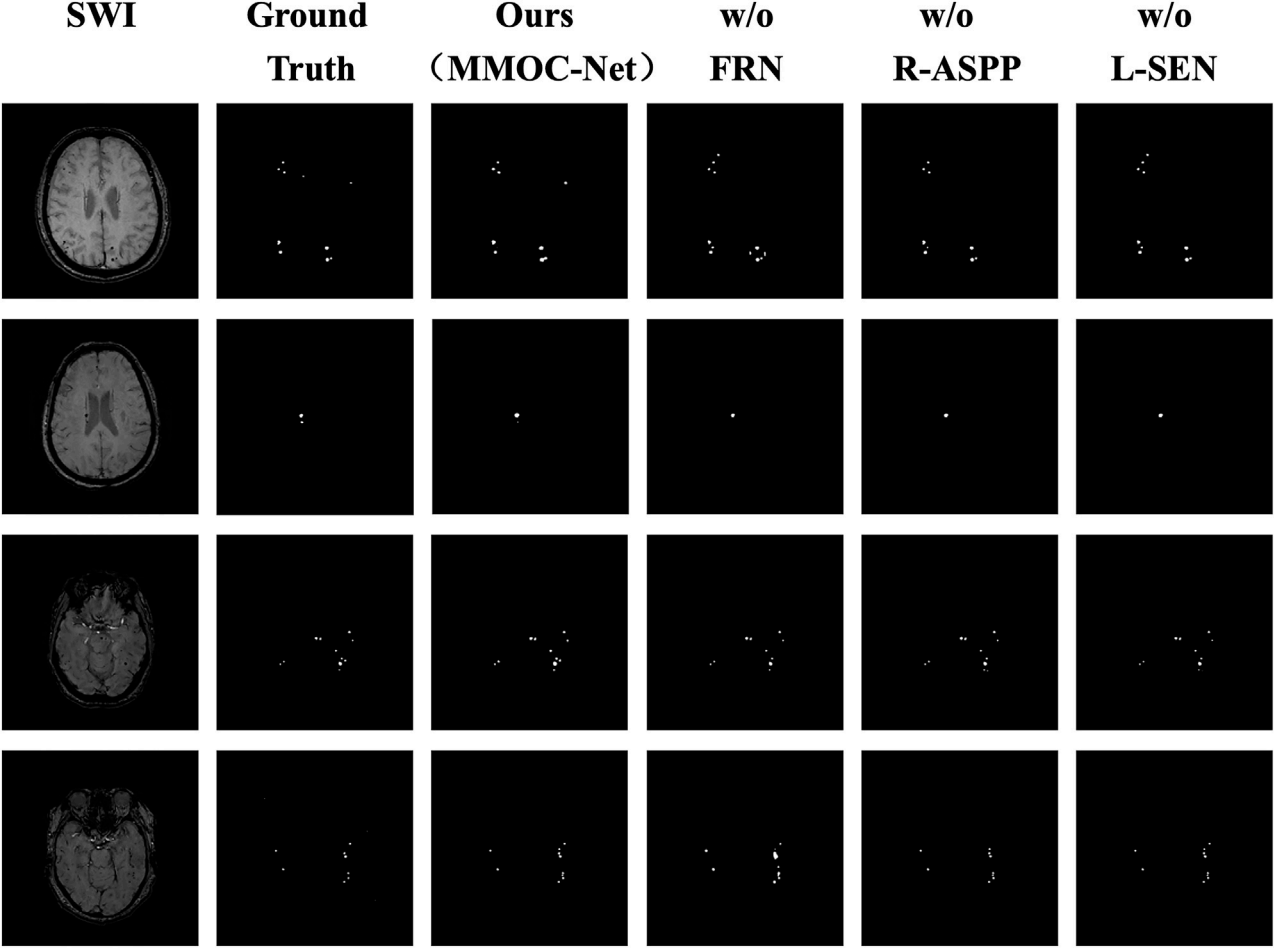
Comparison of the masks of the four ablation combinations. The columns from left to right represent the original image, the ground truth, the MMOC-Net model (ours), w/o FRN, w/o R-ASPP, and w/o L-SEN; the rows from top to bottom represent case Nos. 1–4, respectively. In case 1, the lesion in the upper right corner is correctly predicted by only MMOC-Net, while missed diagnoses and misdiagnoses were obtained using the other three models. In case 2, the small lesion in the lower right corner is correctly predicted only by MMOC-Net, while miss diagnoses occurred using the other three models. In case 3, the number and distribution of lesions are correctly predicted by MMOC-Net, while miss diagnoses occurred using the other three models. In case 4, MMOC-Net most closely approximates the number and distribution of the ground truth, whereas using the other three models obtain missed diagnoses and misdiagnoses.

In this paper, the results of the four ablation combinations are demonstrated, and the segmentation performance results of the different methods are visually compared, as shown in Figure 8. The w/o FRN model has less effective segmentation performance, and more incorrect results are obtained. The w/o R-ASPP model has poor segmentation performance, and key lesion points are easily missed. The SEN value of w/o L-SEN is low, and leakage segmentation appears more frequently. Relative to other ablation combinations, the segmentation profile of MMOC-Net is the closest to the ground truth, indicating higher segmentation accuracy.

### 4.4 Comparison With Previous Studies

Our method aims at the automatic segmentation of CMBs, and its comparison with previous studies can be divided into two aspects. On the one hand, our method is comparable to studies on CMB detection; on the other hand, our method is comparable to studies on segmentation of various objects in the brain.

In deep learning research on CMBs, most previous studies limited research to target detection, and there are few studies on the automatic segmentation of CMBs. Taking CMBs as the research object, we compare our segmentation research with previous detection research. The results show that for the same indicators, such as SEN, PRE, and FP_avg_, better results are achieved in this study than in previous studies, as shown in Table 5. Possible reasons for this discrepancy include differences in data sources, method construction, and mask types. Compared with target detection, the evaluation indicators of segmentation research are more comprehensive, and additional information such as lesion size, location, and volume can be obtained.

**TABLE 5:** Comparison of previous studies on the detection of CMBs and ours.

Research	DSC	SEN	PRE	FP_avg_
Myung et al. (2021)	—	66.90	79.80	2.10
Rashid et al. (2021)	—	84.00	59.00	—
Li et al. (2021b)	—	90.00	76.40	—
Dou et al. (2016)	—	92.26	42.67	2.90
Liu et al. (2019)	—	93.50	75.50	1.63
Al-masni et al. (2020)	—	93.63	61.94	1.42
Ours	**87.93**	**93.87**	**82.69**	**0.16**

Note: “—” means that this evaluation index was not obtained in this study. Bold values represent the best results in the table.

In a comparison of automated segmentation studies (Duan et al., 2020), implemented CMB segmentation in non-SWI sequences, but a DSC of only 50.30% was achieved. Fan et al. (2022) implemented 3D segmentation for CMBs, but a DSC of only 72.00% was achieved. There have also been studies on brain vessels (Dang et al., 2022), haemorrhage strokes (Li et al., 2021a) and white matter hyperintensities (Li et al., 2018). In our study, an excellent performance, with a DSC of 87.93%, is achieved for the automatic segmentation of CMBs, as shown in Table 6.

**TABLE 6:** Comparison of previous brain objects segmentation studies and ours.

References	DSC	F1	SEN	PRE
Duan et al. (2020)	50.30	71.30	—	—
Fan et al. (2022)	72.00	71.80	76.50	71.80
Dang et al. (2022)	79.32	—	—	—
Li et al. (2021a)	80.33	—	—	—
Li et al. (2018)	78.80	77.29	—	—
Ours	**87.93**	**87.93**	**93.87**	**82.69**

Note: “—” means that this evaluation index was not obtained in this study. Bold values represent the best results in the table.

### 4.5 Development of Visualization System

**FIGURE 9:**
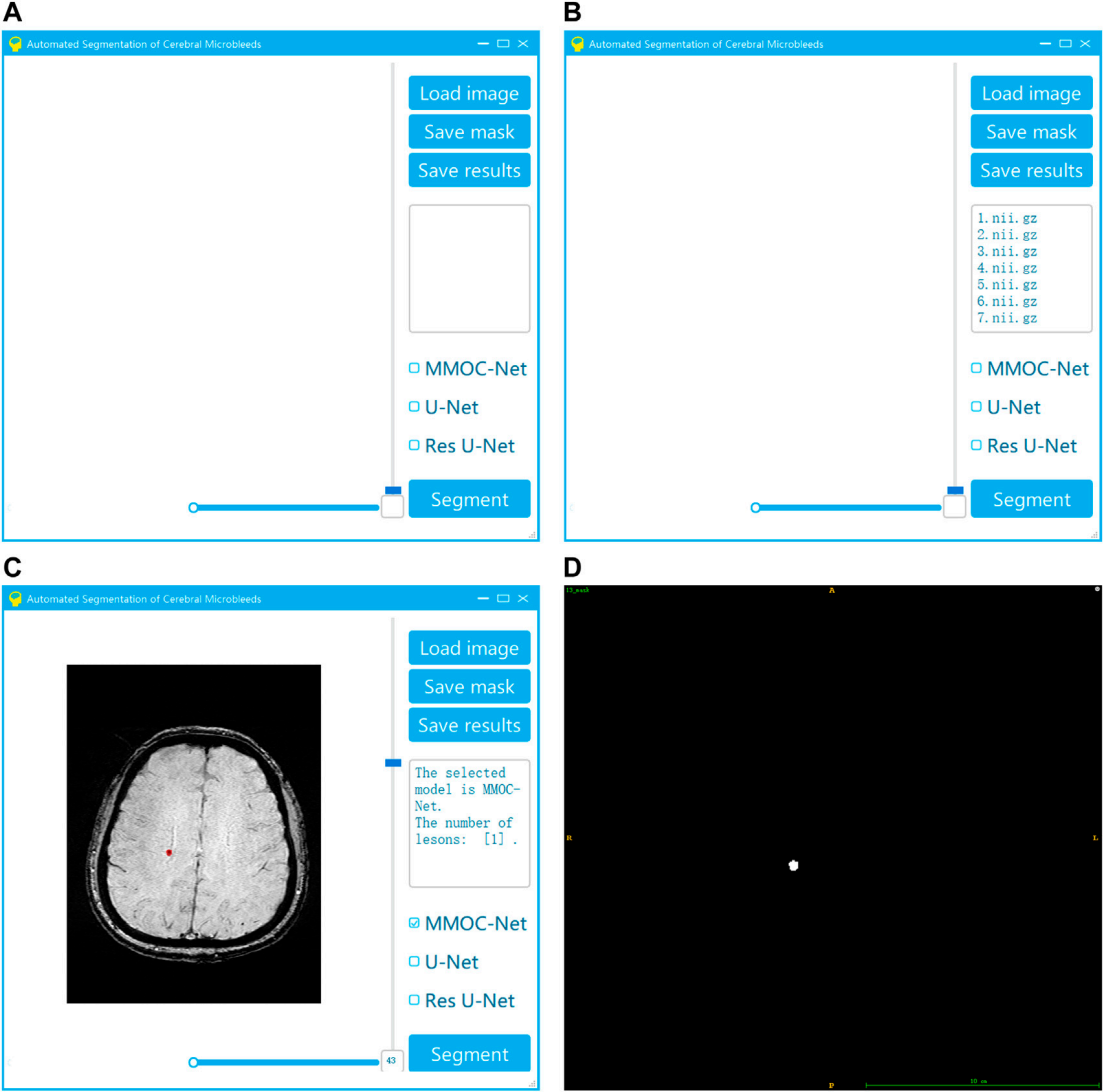
Visualization system for the automatic segmentation of medical small targets. **(A)** Interface of system startup; **(B)** Selection of the original image and segmentation method; **(C)** Running the segmentation to obtain the results; **(D)** Exporting the segmentation results.

To improve the generalization ability of the method in this paper, we also innovatively developed visual automatic segmentation software, as shown in Figure 9. In practical applications, clinicians can efficiently and quickly obtain segmentation results based on deep learning, providing effective support for clinical diagnosis and treatment decisions. Our system has the advantages of convenience, speed and efficiency. After the operator selects the original image and segmentation method, the segmentation result can be automatically output. Using our system, the threshold for physician operation is lowered. As a result, this system can become more popular and may be applied in the majority of primary hospitals.

## 5 Discussion

The segmentation of medical micro-objects is of great significance to the planning of clinical diagnosis and treatment, and it has become a popular issue in recent research. CMBs have been recognized as important biomarkers for the diagnosis of cerebrovascular diseases and the assessment of neurological dysfunction (Greenberg, 2021). However, in clinical practice, manual labelling of CMBs is laborious and diagnosis can easily be missed. There were few previous studies on automated image processing of CMBs, and they only focused on target detection. Few related studies on the automated segmentation of CMBs in SWI have been performed thus far. Automated segmentation of CMBs is beneficial to alleviate the work of physicians and improve the efficiency of diagnosis and treatment. The CMB automated segmentation task is performed for the first time in this study, which has seminal research significance and application prospects.

In this study, we construct a new MMOC-Net model that focuses on the segmentation of clinical microsamples and is implemented in a cascade. To the best of our knowledge, this is the first study to complete automated CMB segmentation in SWI sequence, which utilizes a new deep learning technique to efficiently analyze image information. In the cascade architecture, the first stage focuses on excluding background regions and screening potential candidate regions. We have developed a modified U-Net model for this stage, and the impact of the SEN in the modified loss function is valued. The second stage focuses on a small number of candidates and excludes false-positive regions with similar appearances to CMBs. At this stage, we employ FRN integrated with R-ASPP to identify CMBs. This results in higher SEN values and lower false-positive rates, which can better meet the requirements of accuracy.

In comparison with previous studies, the segmentation performance of MMOC-Net is excellent. This performance is better than the performance of similar studies of previous target detection and segmentation. The results of this study confirm that a properly trained MMOC-Net can achieve the accuracy of experienced clinicians and greatly improve the efficiency of diagnosis and treatment.

Compared with previous segmentation studies, the advantages of our method are as follows: 1) We adopt the implementation of coarse to fine segmentation, and FRN integrated with the R-ASPP module is beneficial for extracting full-resolution, multiscale image features, efficiently detecting CMB lesions. The above measures can consider global and local information and can achieve better feature extraction accuracy. 2) We propose a modified loss function, creatively add the optimization goal of sensitivity, and adopt different weight settings in the two-stage training, which is beneficial to reduce the missed diagnosis rate as much as possible on the premise of ensuring a certain precision. 3) In this paper, the ablation study is used to determine the optimal ROI size. With a size of 32 × 32, global and local information can be balanced well, and better feature extraction accuracy can be achieved.

Compared with previous research on object detection, there are several possible reasons as to why our method, which focuses on segmentation, can still achieve relative advantages: 1) Method: In our method, innovative improvements have been made in the network structure, parameter settings and objective function. 2) Label type: There are differences in annotations between detection and segmentation; detection uses bounding box labels, while segmentation uses fine-grained pixel-level labels. Compared with other detection methods, we use pixel-level accurate data for training and achieve better prediction results. 3) Information mining: The evaluation metrics for the accurate segmentation of CMBs generated by our method are more comprehensive and can provide more information, such as distribution, quantity, and size, which can be combined with relevant research on risk factors to provide effective support for clinical diagnosis and treatment decisions (Whitwell et al., 2015).

In practical applications, this method has high clinical significance. 1) Automation: In clinical screening and diagnosis, manual detection and segmentation of CMBs is an expensive, tedious and time-consuming task. Rapid and accurate automated CMB segmentation can alleviate the burden of clinicians so that they can focus on higher-level clinical decisions. 2) Effectiveness: CMB segmentation is highly skill- and experience-demanding for clinicians, and the labelling accuracy of different clinicians varies greatly. For example Duan et al. (2020), showed that the DSC of clinicians with many years of experience in manually segmenting CMBs is only 57.60%. In contrast, our method is an automated and standardized segmentation approach based on deep learning, which can effectively improve the segmentation accuracy and reduce the risk of missed diagnosis. 3) Visualization: We have also innovatively developed a visual automatic segmentation system, which has the advantages of convenience, speed and efficiency. Our system is conducive to lowering the threshold for physicians to operate. This can be popularized and applied in the majority of primary hospitals. 4) Generalizability: The existence of CMBs is related to a variety of cerebrovascular disease accidents and death risks. After acquiring CMB segmentation regions by the method in this paper, clinical information can be extracted from the distribution, quantity and size of CMBs to provide suggestions for further clinical interventions for patients.

Our study was well designed and rigorously implemented, but there were still some deficiencies and room for improvement. We envision future research directions as follows. 1) This study focuses on 2D image segmentation without utilizing 3D image features. In the future, we need to implement 3D layer segmentation to better utilize the spatialized, stereologic information of SWI images. In addition, the present study is a single-centre study with a relatively limited sample size, and it lacks validation on external datasets. In the future, we need to include more datasets with small medical micro-objects and perform studies with large sample sizes and multicentre validation. 2) There is still room to improve the segmentation performance of this method, and the generalization performance of the newly constructed MMOC-Net needs to be verified. In the future, we need to continue to optimize the relationship between the model structure and model parameters, improve the segmentation performance, and better apply the model in clinical diagnosis and treatment decisions.

## 6 Conclusion

In summary, we perform the automatic segmentation task of CMB images in SWI and create a brand-new segmentation method (MMOC-Net) with pioneering research implications. MMOC-Net can improve the diagnosis and treatment efficiency while reducing the missed diagnosis rate and can obtain a better segmentation performance (87.93% DSC and 90.69% F2). Our idea and method can realize the target detection and fine segmentation of medical micro-objects, which can make full use of image information to provide clinical decision support and has great application value and promotion prospects.

## Data Availability

The datasets presented in this study can be found in online repositories. The names of the repository/repositories and accession number(s) can be found below: https://github.com/qweiweili/MMOC-Net.

## Abbreviations

CMBs, cerebral microbleeds; CSVD, Cerebral small vessel disease; DSC, Dice similarity coefficient; F2, F2 score; FRN, full-resolution network; MMOC-Net, medical micro-object cascade network; PRE, precision; R-ASPP, atrous spatial pyramid pooling; SEN, sensitivity; SWI, susceptibility weighted imaging; ROI, region of interest.
